# Book Review: Student Engagement: Effective Academic, Behavioral, Cognitive, and Affective Interventions at School

**DOI:** 10.3389/fpsyg.2021.711932

**Published:** 2021-06-11

**Authors:** Feiya Hou, Dan Li

**Affiliations:** School of Foreign Languages, Beihua University, Jilin City, China

**Keywords:** student engagement, interventions, school, learning strategies, contemporary education

Echoed as the “the holy grail of learning” (Sinatra et al., 2015, p. 1), engagement has been one of the most prevailing research areas in contemporary education, which has piqued the interest of many scholars from different disciplines. It is generally postulated that student engagement has four pivotal characteristics; it is action-based, context-specific, object-oriented, and dynamic (Reschly and Christenson, 2012). Therefore, a learner's engagement does not happen in a vacuum; it dynamically emerges as a result of the interaction with families, schools, peers, classrooms, cultures, activities, as well as tasks which *per se* impact multifarious layers of engagement and learning emotionally, academically, cognitively, and behaviorally. As such, it is vivid why student engagement has gained significant momentum among scholars and practitioners. One such surge of interest is instantiated in *Student Engagement: Effective Academic, Behavioral, Cognitive, and Affective Interventions at School*, edited professionally by Amy L. Reschly, Angie J. Pohl, and Sandra L. Christenson who are distinguished scholars in studies on student engagement. This compendium provides evidence-based strategies and interventions that aim at targeting students' engagement at school. This volume is the outcome of a 29-year history of the Check & Connect Model that aims to (a) foreground the engagement subtypes that underscore Check & Connect and (b) explore rigorously based interventions that boost student engagement subtypes, so it is a much-to-be-welcomed contribution.

Structurally, the book encompasses five parts, organized thematically into 16 chapters. Part I, comprising four chapters, focuses on the relevance of student engagement and the impact of implementing Check & Connect (Chapter 1), presents the evolution of the student engagement construct and school dropout (Chapter 2), exemplifies a wide range of techniques that can measure students' academic, behavioral, cognitive, as well as affective engagement (Chapter 3), and delineates how treatment fidelity, as an evidence-based intervention, can be applied to enhance implementer success and boost student outcomes.

Part II, entitled Academic Engagement, includes three chapters. Chapter 5 conceptualizes academic engagement, justifies its significance in student achievement, and provides different ways to promote it. Chapter 6 reports on the findings of Peer-Assisted Learning Strategies (PALS) as a classroom-based intervention to strengthen reading and math outcomes which leads to an academic safety-net for students. Being cognizant of the pivotal role of homework in success and engagement, the authors in Chapter 7 highlight the importance of the Homework, Organization, and Planning Skills (HOPS) intervention for engaging students in the learning process.

Part III, Behavioral Engagement, contains four chapters. Chapter 8 defines academic engagement, justifies its significance in student achievement, and offers different interventions to promote it (see Table 8.2). Chapter 9 elucidates how learners' behavioral engagement can be optimized through the Good Behavior Game, an adaptable and acceptable model to promote positive classroom learning environments, which accentuate “group contingency classroom behavior management and instructional support approach” (226). In Chapter 10, the authors flesh out how a system of support rooted in a solid foundation can increase students' engagement in school settings. They postulate that School-Wide Positive Behavioral Interventions and Supports (SWPBIS) can systematically increase behavioral engagement. What seems genuinely engaging in Chapter 11 is to see how Check-In, Check-Out (CICO), an evidence-based intervention, is influential in bolstering the majority of students' involvement.

Part IV, Affective Engagement, encompasses two chapters. Chapter 12 defines affective engagement, enumerates its core components, and stipulates that affective engagement, as a multitiered and dynamic dimension of engagement, is amenable to prevention and intervention enterprises. The other chapter in this section takes advantage of Banking Time as another intervention technique to engage students in the process of learning and teaching emotionally. The authors cogently argue “*Banking Time* is an affective intervention that evidences good usability, transportability, and sustainability within the school context” (p. 342–343).

Part V, Cognitive Engagement, includes 3 chapters. Chapter 14 brings to the fore the importance of cognitive strategies and interventions in students' learning and mental engagement. The penultimate chapter explicates the theoretical, instructional, and empirical foundations for another intervention program, called Self-Regulation Empowerment Program (SREP), to engage students cognitively. Since SREP is still in its infancy, the author convincingly calls for some more empirical studies to be conducted on the applicability of this program across different grades and levels. In the closing chapter, the authors adroitly touch upon growth mindset as an insightful topic to promote cognitive engagement. The authors postulate that students' cognitions about their ability, intelligence, and effort improve their growth mindset and provide some viable strategies to enhance their cognitive engagement.

This compendium offers a number of thought-provoking insights and lessons. First, inasmuch as the fact that student engagement is not a mono-dimensional phenomenon, the editors have sagaciously incorporated all the dimensions, including academic, affective, behavioral, and cognitive, in this volume. Secondly, the volume is replete with theoretically rich, methodologically rigorous, and pedagogically convincing chapters which can be applied in different contexts to minimize school dropout and maximize student achievement. Thirdly, the validated instruments utilized and suggested in some of the chapters can be a treasure-trove for other researchers to embark on them in their own contexts. Nonetheless, we expected that the editors include one or two chapters on how student engagement can be defined and operationalized in cross-cultural studies. Besides, scrutinizing developmental and longitudinal trajectories of student engagement remains scanty.

All in all, this enlightening and groundbreaking compendium is a must-have source that remains contemporary for a long time for professionals, researchers, graduate students, education policymakers, and government advisors on education and human development.

## Author Contributions

FH and DL both contributed to the design and writing of the work.

## Conflict of Interest

The authors declare that the research was conducted in the absence of any commercial or financial relationships that could be construed as a potential conflict of interest.
